# Ratio of IL-8 in CSF Versus Serum Is Elevated in Patients with Unruptured Brain Aneurysm

**DOI:** 10.3390/jcm9061761

**Published:** 2020-06-05

**Authors:** Joanna Kamińska, Tomasz Lyson, Robert Chrzanowski, Karol Sawicki, Anna J. Milewska, Marzena Tylicka, Justyna Zińczuk, Joanna Matowicka-Karna, Violetta Dymicka-Piekarska, Zenon Mariak, Olga M. Koper-Lenkiewicz

**Affiliations:** 1 Department of Clinical Laboratory Diagnostics, Medical University of Białystok, Waszyngtona 15A St., 15-269 Białystok, Poland; justyna.zinczuk@umb.edu.pl (J.Z.); matowic@umb.edu.pl (J.M.-K.); violetta.dymicka-piekarska@umb.edu.pl (V.D.-P.); 2 Department of Neurosurgery, Clinical Hospital of the Medical University of Białystok, M. Skłodowskiej-Curie 24A St., 15-276 Białystok, Poland; lyson_t@vp.pl (T.L.); robert.e.chrzanowski@gmail.com (R.C.); sawicki_karol@o2.pl (K.S.); zmariak@umb.edu.pl (Z.M.); 3 Department of Statistics and Medical Informatics, Medical University of Bialystok, ul. Szpitalna 37, 15-295 Białystok, Poland; anna.milewska@umb.edu.pl; 4 Department of Biophysics, Medical University of Białystok, Mickiewicza 2A St., 15-089 Białystok, Poland; marzena.tylicka@umb.edu.pl

**Keywords:** cerebrospinal fluid, IL8/CXCL8–interleukin 8/C-X-C motif chemokine ligand 8, MCP-1/CCL2–monocyte chemoattractant protein-1/C-C motif ligand 2, unruptured intracranial aneurysm (UIA)

## Abstract

Only scarce data pertaining to interleukin 8 (IL-8) and monocyte chemoattractant protein-1 (MCP-1) chemokines in human aneurysm can be found in the current literature. Therefore, the aim of this study was the evaluation of cerebrospinal fluid (CSF) and serum IL-8 and MCP-1 concentration in unruptured intracranial aneurysm (UIA) patients (*n* = 25) compared to the control group (*n* = 20). IL-8 and MCP-1 concentrations were measured with ELISA method. We demonstrated that CSF IL-8 concentration of UIA patients is significantly higher (*p* < 0.001) than that presented in the serum, which can indicate its local synthesis within central nervous system. CSF IL-8 concentration was also significantly related to aneurysm size, which may reflect the participation of IL-8 in the formation and development of brain aneurysms. IL-8 Quotient (CSF IL-8 divided by serum IL-8) in UIA patients was statistically higher compared to control individuals (*p* = 0.045). However, the diagnostic utility analysis did not equivocally indicate the diagnostic usefulness of the IL-8 Quotient evaluation in brain aneurysm patients. Nevertheless, this aspect requires further study.

## 1. Introduction

Intracranial cerebral aneurysms (IAs) occur in approximately 5% of the population [1,2]. Risk factors include: hypertension, atherosclerosis, smoking, alcohol consumption, female sex, and genetic predisposition [3,4,5,6,7]. Cerebral aneurysms are usually asymptomatic but associated with the risk of a life-threatening intracranial hemorrhage [8]. IAs are most often diagnosed only incidentally during magnetic resonance imaging (MRI) or computed tomography (CT) performed for a variety of reasons, like headaches or head injuries. However, it must be stressed, that a good portion of small aneurysms can escape visualization with standard MRI/CT, while still retaining potential for rupture and bleeding [9]. Unruptured intracranial aneurysms (UIAs) with a high estimated risk of rupture (i.e., of unfavorable location, size and structure) are secured by surgical clipping or by intravascular embolization. The UIAs with low estimated probability of rupture are often not qualified for obliteration because of the risk inherent to surgical intervention [10,11,12,13].

At present, both primary diagnosis of IAs and follow up of those treated conservatively rely exclusively on their visualization. Nevertheless, the imaging either with MRI or with CT is hampered by high costs, limited availability, and a degree of biohazard related to irradiation and the use of a contrast agent [14]. Therefore, any method of screening for the IA’s in population and/or for assessing the dynamics of aneurysm growth would be of paramount importance. Unfortunately, such methods today are in short supply (if at all available), thus giving incentive for the prompt identification of biological markers of IA’s development.

Chemokines are a heterogeneous group of soluble, short-acting proteins and peptides that are strong mediators of the inflammatory response [15,16]. Animal studies indicate two chemokines—C-X-C motif chemokine–ligand–8 (CXCL8/IL-8) and monocyte chemoattractant protein-1 (CCL2/MCP-1)—especially important in the formation and rupture of cerebral aneurysms [17,18,19]. IL-8 belongs to the CXC family of chemokines possessing the ELR motif (Glu-Leu-Arg), attracting mainly neutrophils [20,21]. It is secreted by various cell types—monocytes, macrophages, fibroblasts, epithelial cells—but also vascular endothelial cells [22,23]. MCP-1 is a potent chemoattractant for macrophages, T lymphocytes, NK cells and basophils [24,25]. It is expressed by the endothelial cells of the aneurysm under high wall shear stress, which promote macrophage infiltration at an early stage of brain aneurysm formation [17,26].

In contrast to the animal models, there are only scarce data pertaining to aneurysms in humans [27,28]. It should be also noted that studies carried out on clinical material of aneurysms after subarachnoid hemorrhage (SAH) may result in rather confusing conclusions as to the problem of primary aneurysm formation and growth [29]. The hitherto published studies were based on sampling blood for the presence of the cytokines. A question needing answering is whether local chemokines’ concentration around the aneurysm is reliably represented by their concentration in blood. An answer can be brought forth only by evaluation of the concentrations in the cerebrospinal fluid (CSF) and in the peripheral blood.

Taking into consideration the above, the aim of the study was the evaluation of CSF and serum IL-8 and MCP-1 concentration in patients with unruptured intracranial aneurysm. To exclude influence of the blood–brain barrier and the blood–CSF barrier on mutual relationships of protein levels in these two compartments [30,31,32], we also calculated IL-8 and MCP-1 Quotients by referring their CSF values to the serum values. In our study we tested if the evaluation of IL-8 and MCP-1 concentration would allow to diagnose brain aneurysm before its rupture. We also attempted to check if IL-8 and MCP-1 levels or their Quotients are related to aneurysm size, number and shape, as well as aneurysm risk factors (age, gender, smoking, hypertension, obesity).

## 2. Material and Methods

### 2.1. Subjects

The study was conducted in agreement with the Helsinki-II-declaration and the protocol was approved by the Bioethics Human Research Committee of the Medical University of Bialystok (Permission No. R-I-002/383/2015). All subjects gave their informed consent for inclusion before they participated in the study.

The study group consisted of 25 patients: five males/20 females, mean age 56 years, range 30–70 years. All of them were subjected to craniotomy and direct surgical clipping of unruptured intracranial aneurysm and were operated on by one surgeon (Z.M.). In all patients, their aneurysms were asymptomatic and located at the anterior part of the Willis circle. A decision regarding the need for intervention was undertaken after careful consideration of the risk of aneurysm rupture versus inherited risk of surgical procedure. The commonly accepted guidelines (e.g., size > 5 mm and/or irregular shape, representing increased risk of rupture [12] and the opinion of the patient) were taken into consideration when making the decision. The presence of UIA was established with CT angiography in 14 patients and with MR angiography in 11 patients. In eight individuals, a precise definition of the location, size and shape of the aneurysm required confirmation with contrast digital angiography.

Exclusion criteria included: neurodegenerative conditions like multiple sclerosis, neuroinfection and brain tumor in medical history, surgery or major trauma in the previous months, anti-inflammatory, antibiotics, or corticosteroids administration in the previous months.

The comparative group was composed of 20 subjects (six males/14 females, mean age 57 years, range 25–78 years), suffering from trigeminal neuralgia due to anatomical conflict between the trigeminal nerve and a cerebellar artery. All patients belonging to this group revealed refractory to conservative treatment and were qualified for posterior fossa craniotomy and microvascular decompression. The procedure involved exposition of the conflict at the cerebro-pontine angle, desinsertion of the compressing artery from the trigeminal nerve, displacement of the artery, and finally its fixation to the cerebellar tentorium with a sticking material (Tachosil^®^, Takeda, Linz, Austria). The criteria of exclusion from the control group were exactly the same as observed in the study group.

All patients before surgery had fasting basic laboratory tests done between 6:00 and 7:00 a.m. Table 1 and Table 2 present the characteristics of the study and control subjects. Tobacco intake was defined when patient smokes more than 20 cigarettes a day for over 12 months and more. People with body mass index (BMI) > 30.0 (kg/m^2^) were classified as obese.

### 2.2. Sample Collection and Storage

CSF was obtained during craniotomy from the subarachnoid space of the brain. Surgical procedures were performed in a standard manner: under general anesthesia and with the patient’s head fixed in a three-pin Mayfield headholder. Skin incision preceded the lifting of the bone flap and lancing of dura mater, which allowed visualization of the arachnoid membrane and subarachnoid space. With the aid of an operating microscope, the subarachnoid space was carefully opened and inflowing CSF aspirated with single-use, sterile syringe, and soft venous catheter. Particular care was taken to prevent any contamination of the CSF with blood and with warm saline solution used as irrigation. Therefore, all the aforementioned steps were taken in the very beginning of each procedure, before any bleeding may occur.

Patients’ blood samples (2.7 mL) were collected into test tubes without anticoagulant (S-Monovette, SARSTEDT, Nümbrecht, Germany). Within 0.5 h after the venipuncture, blood was centrifuged for 20 min at 1000× *g* to obtain serum. CSF samples were centrifuged for 20 min at 1000× *g*. Obtained serum and CSF supernatant were aliquoted and stored at −75 °C until further analysis.

### 2.3. IL-8 and MCP-1 Concentrations Evaluation

Cerebrospinal fluid (CSF) and serum IL-8 and MCP-1 concentrations were measured using enzyme-linked immunosorbent assay (ELISA) tests: ELISA Quantikine^®^ Human CXCL-8/IL-8 Immunoassay kit (Catalogue number: D8000C) and ELISA Quantikine^®^ Human CCL2/MCP-1 Immunoassay kit (Catalogue number: DCP00), both from R&D Systems Europe Ltd., Abingdon, England. Experiment was conducted in accordance with the manufacturer’s instruction. CSF and serum samples for IL-8 evaluation were not diluted prior to analysis. The manufacturer of the assay kit referred to the intra-assay coefficient of variation (CV%) as 5.6% at IL-8 mean concentration of 168 pg/mL, SD = 9.4 pg/mL. CSF and serum samples for MCP-1 evaluation were diluted 2-fold prior analysis. The manufacturer of the assay kit referred to the intra-assay coefficient of variation (CV%) as 7.8% at MCP-1 mean concentration of 76.7 pg/mL, SD = 6.0 pg/mL.

To calculate IL-8 and MCP-1 Quotients, we divided CSF chemokine result by its concentration in serum, as described elsewhere [30,31,32]. Such an approach allowed to exclude possible influence of the blood–CSF barrier impairment and/or blood–brain barrier functions on IL-8 and MCP-1 levels.

### 2.4. Statistical Analysis

The obtained results were analyzed with the use of the STATISTICA 12.0 PL software (StatSoft Inc., Tulsa, OK, USA). The distribution of the studied concentrations did not follow the normal distribution (X^2^-test), thus nonparametric statistical analyses were applied. The Mann–Whitney test was used in order to compare two independent samples. The X^2^-test with Yates correction was used to investigate whether patients were gender-matched and whether there were differences regarding risk factors present between them. Correlation coefficients were obtained by applying Spearman’s rank correlation. Values for continuous variables are presented as median with the 25th and 75th percentiles. For the variables CSF and serum chemokines concentration, chemokines Quotients, age, sex, and brain aneurysm risk factors, a logistic regression model was sought. The same covariables were used in the linear regression analysis of predictor variables influencing aneurysm size as the ones in the logistic regression analysis. Differences were considered statistically significant at *p* < 0.05. Receiver operator characteristic (ROC) curve was generated in order to determine the performance of cytokine evaluation in a task of discrimination between the study and control groups. The Youden index, a function of sensitivity and specificity, indicated an optimal trade-off between these two (cut-off point) for the parameters tested.

## 3. Results

### 3.1. IL-8 Results

**Figure 1:**
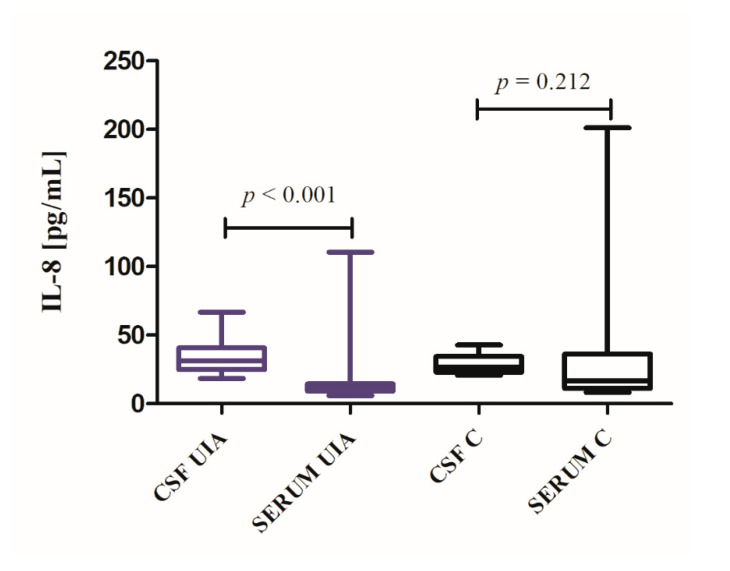
IL-8 cerebrospinal fluid (CSF) and serum concentrations in unruptured intracranial aneurysm (UIA) patients compared to control group (C).

CSF IL-8 concentration was higher, while serum IL-8 concentration lower in UIA patients compared to the control group, but differences were not significant (see Supplementary Materials: Table S1) In UIA patients, CSF IL-8 median concentration was 3-fold higher compared to median serum concentration (*p* < 0.001). In the control group, CSF IL-8 was 1.6-fold higher compared to median serum concentration, but the obtained difference was not significant (*p* = 0.212) (Figure 1).

**Figure 2:**
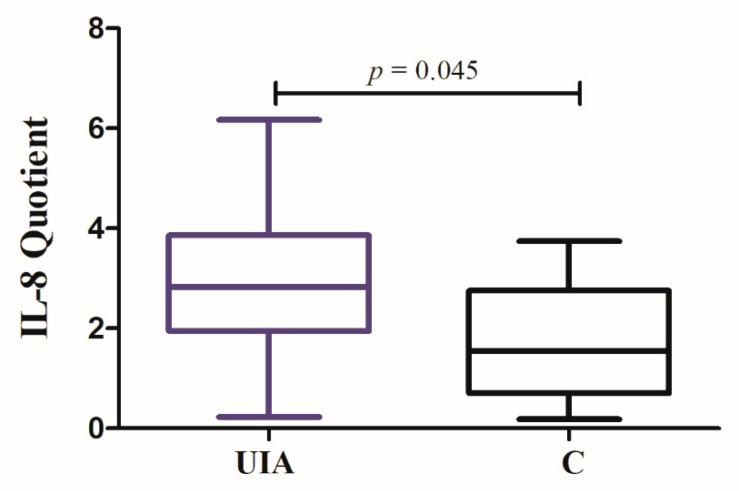
IL-8 Quotient in unruptured intracranial aneurysm (UIA) patients compared to control group (C).

**Figure 3:**
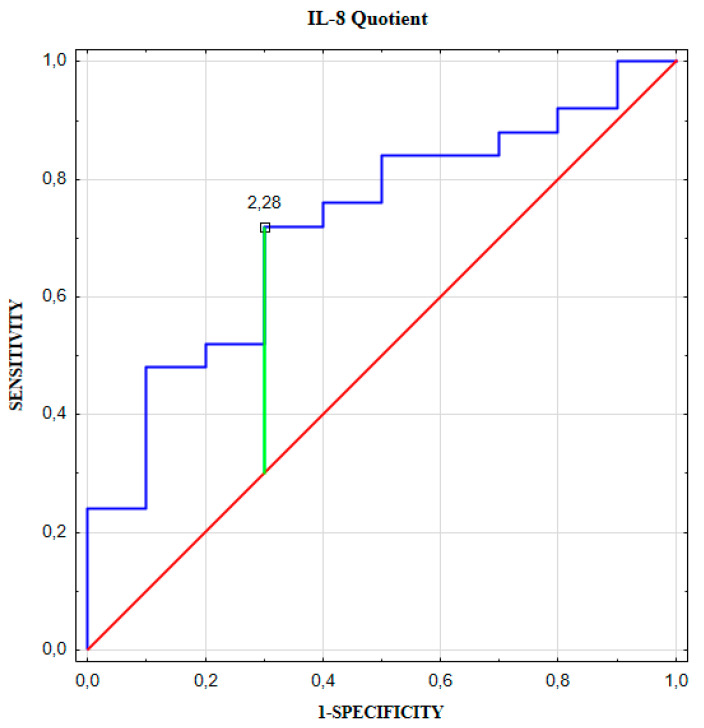
IL-8 Quotient receiver operator characteristic (ROC) curve for differentiating unruptured intracranial aneurysm patients from individuals without brain aneurysm.

IL-8 Quotient in UIA patients was statistically higher compared to control individuals (Figure 2**,** see Supplementary Materials: Table S1). The area under the ROC curve (AUC) for IL-8 Quotient was statistically higher than the value of 0.5 (Figure 3, Table 3).

### 3.2. MCP-1 Results

**Figure 4:**
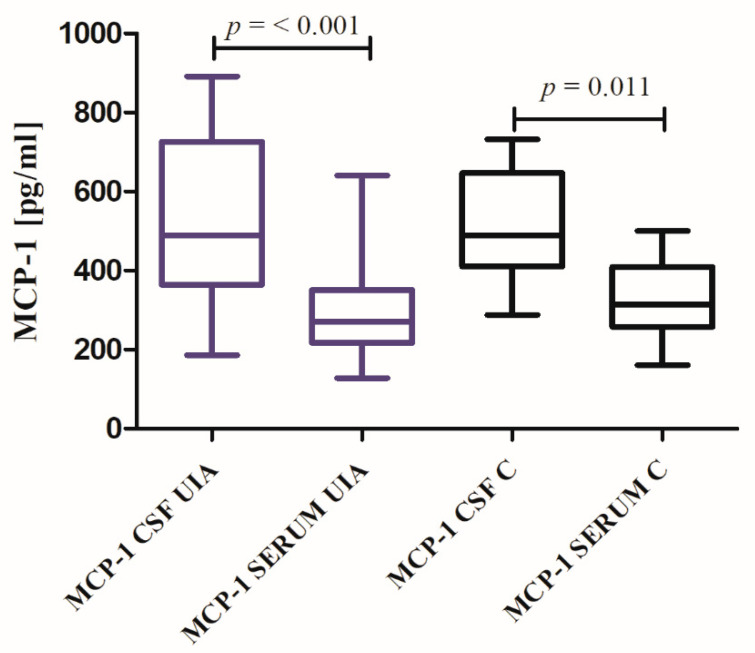
MCP-1 cerebrospinal fluid (CSF) and serum concentrations in unruptured intracranial aneurysm (UIA) patients compared to control group (C).

**Figure 5:**
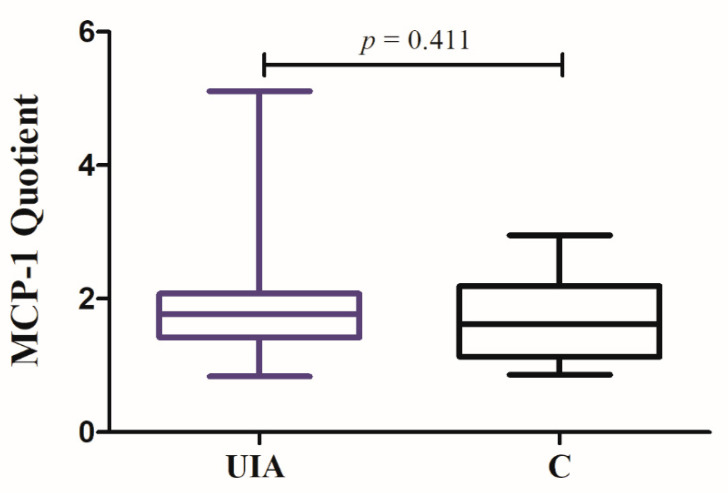
MCP-1 Quotient in unruptured intracranial aneurysm (UIA) patients compared to control group (C).

The CSF MCP-1 concentration was similar, while serum MCP-1 concentration was somewhat lower in UIA group compared to the control individuals, but both differences were not statistically significant (see Supplementary Materials: Table S1). In UIA patients as well as in the control group, CSF MCP-1 median concentration was statistically higher (1.8-fold, 1.6-fold, respectively) compared to median serum concentration (*p* < 0.001, *p* = 0.011, respectively) (Figure 4). The MCP-1 Quotient showed a tendency to be higher in UIA patients compared to control group, but again, the difference was not significant (Figure 5, see Supplementary Materials: Table S1).

### 3.3. Logistics Regression Analysis Results

For the variables CSF IL-8 concentration, serum IL-8 concentration, IL-8 Quotient, CSF MCP-1 concentration, serum MCP-1 concentration, MCP-1 Quotient, age, sex, and brain aneurysm risk factors (obesity, systolic blood pressure, diastolic blood pressure, and smoking) a multivariate logistic regression model was sought, but nothing of significance was discovered. Only a univariate linear regression model was obtained. We showed that if the IL-8 Quotient increases by 1, the chance of having unruptured brain aneurysm increases by 1.84 times (increases by 84%) (Table 4).

### 3.4. Correlation Coefficient Results

In UIA individuals, correlation coefficient analysis revealed: positive correlation between CSF IL-8 concentration and aneurysm size (*R* = 0.41, *p* = 0.04), and positive correlation between both CSF and serum MCP-1 concentrations and the aneurysms number (*R* = 0.43, *p* = 0.032 and *R* = 0.40, *p* = 0.049, respectively).

### 3.5. Linear Regression Analysis Results

Logarithmic transformation of aneurysm’s size was necessary for the linear regression model assumptions to be met. The same covariables were used as the ones in the logistic regression analysis. Univariate linear regression analysis revealed that: (1) with an increase in IL-8 concentration in CSF by 10 pg/mL, the aneurysm size increases by 1.14 times (rises by 14%); (2) with an increase in BMI by 1, the aneurysm size increases by 1.035 times (rises by 3.5%) (Table 5).

In the model of multivariate linear regression analysis, predictor variables influencing aneurysm size included: CSF IL-8 concentration and BMI. Adjusted R square (R^2^) for the created model equals 0.39, which indicates that this model explains 39% of the variance in dependent variable. Multivariate linear regression analysis results for brain aneurysm size revealed that: (1) with an increase in IL-8 concentration in CSF by 10 pg/mL, the aneurysm size increases by 1.15 times (rises by 15%); (2) with an increase in BMI by 1, the aneurysm size increases by 1.03 times (rises by 3%) (Table 5).

Patients with aneurysm size ≥ median value (5.4 mm) had statistically higher CSF IL-8 levels compared to those with aneurysm size < median value (Mann–Whitney test, *p* < 0.05). Interestingly, patients with multiple brain aneurysm had higher CSF MCP-1 levels compared to individuals with single aneurysm (Mann–Whitney test, *p* < 0.05, Table 6).

**Figure 6:**
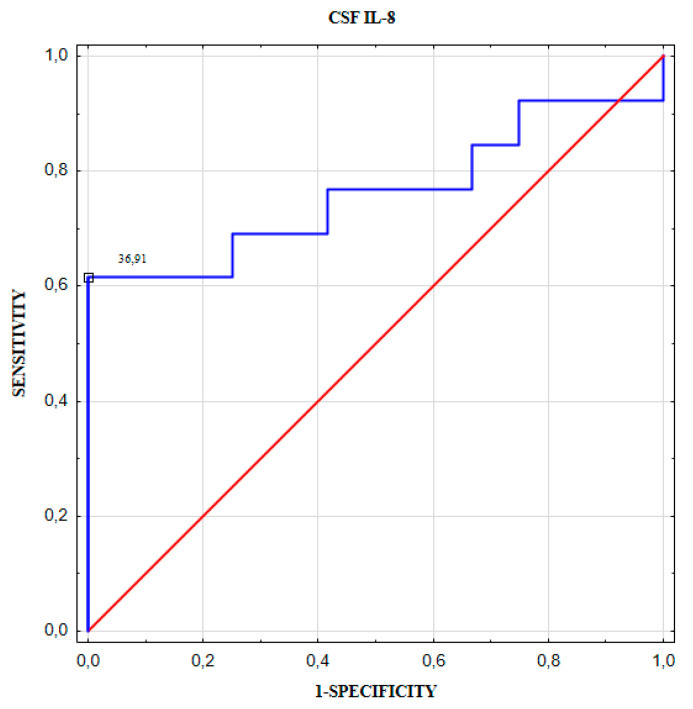
CSF IL-8 receiver operator characteristic (ROC) curve for predicting a larger brain aneurysm size (≥5.4 mm).

**Figure 7:**
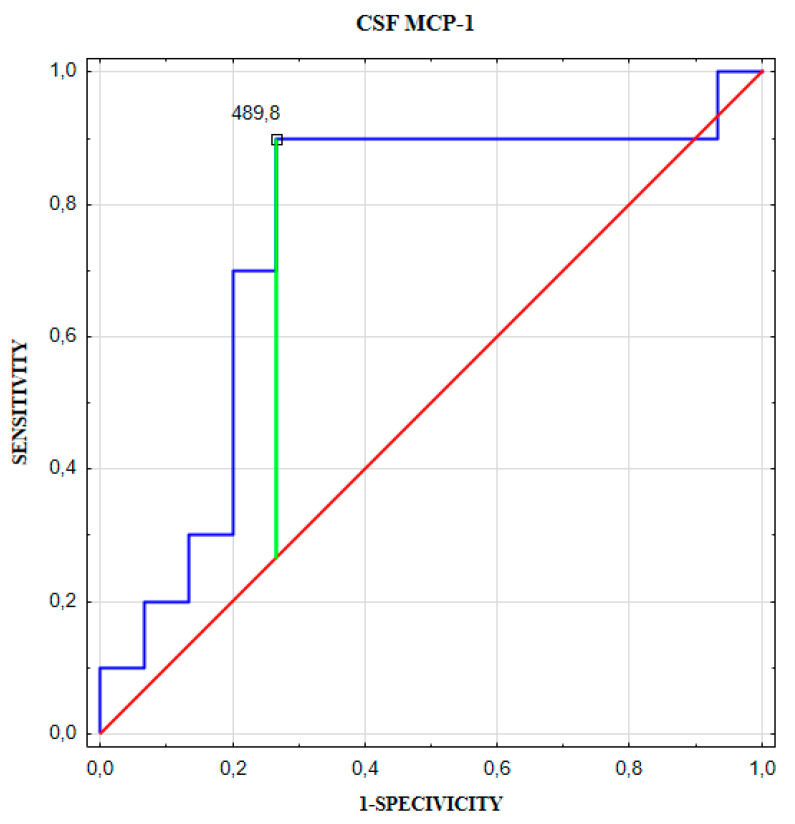
CSF MCP-1 receiver operator characteristic (ROC) curve for predicting a number of aneurysms.

To indicate the optimal cut-off points of chemokines levels to predict the aneurysm size (<5.4 mm versus ≥5.4 mm) and to predict the number of aneurysms (single versus multiple) we performed a ROC curve analysis in the next step. The optimal cut-off point for CSF IL-8 concentration to predict a larger brain aneurysm size (≥5.4 mm) was 36.9 pg/mL (Table 7, Figure 6). The optimal cut-off point for CSF MCP-1 concentration to predict a number of aneurysms was 489.8 pg/mL (Table 8, Figure 7).

As to the “risk factors”, our results suggest that patients with arterial hypertension had significantly lower CSF MCP-1 concentration compared to individuals without hypertension (Mann–Whitney test, *p* < 0.01, see Supplementary Materials: Table S2).

## 4. Discussion

Our study is the first to demonstrate that IL-8 concentration in the cerebrospinal fluid of patients with unruptured intracranial aneurysm is significantly higher than this present in serum (almost 3-fold). In the control group, we did not observe significant differences between these two compartments. These results indicate a local IL-8 synthesis in brain aneurysm patients [33,34] and may reflect the participation of IL-8 in formation and development of brain aneurysm. Especially, we found a correlation between CSF concentration of IL-8 and aneurysm size. This notion is strengthened by multivariate linear regression analysis results indicating that aneurysm size increases with an increase in IL-8 concentration in CSF.

Moreover, we found a weak but positive correlation between MCP-1 concentration in the CSF and the number of aneurysms. Our research shows that the assessment of these chemokines concentration in CSF and serum may be also useful in stratifying the risk of a “dangerous” brain aneurysm presence.

Simultaneous evaluation of the chemokines both in CSF (which abuts the aneurysm), and serum is the advantage of our study. The calculation of protein Quotient eliminates the influence of biological factors like age, gender, infection, which can change the content of blood cytokines. Additionally, such approach minimizes the impact of methodological factors because chemokines are measured in one analytical process [33,34].

We are the first to calculate Quotients for the IL-8 and MCP-1 chemokines in patients with unruptured brain aneurysm. We observed that the Quotient for IL-8 was significantly higher in patients with an unruptured intracranial aneurysm compared to those without vascular lesions in the brain. It is also worth mentioning that IL-8 Quotient tended to increase in patients with larger, polycyclic aneurysms and in patients over 60 years of age, smokers and obese. Univariate logistic regression analysis showed that if the IL-8 Quotient increases by 1, the chance of having unruptured brain aneurysm increases by 84%. Unfortunately, we did not obtain a significant multivariate logistic regression model for IL-8 Quotient.

To assess diagnostic utility of the IL-8 Quotient calculation, we constructed an ROC curve for ex post discrimination between our patients with and without cerebral aneurysm. The area under the curve (amounting to 0.72) corresponds to a moderate overall diagnostic accuracy of the test [35]. The positive predictive value (PPV) and negative predictive value (NPV) of IL-8 Quotient equaled 86% and 50%, respectively. Thus, the NPV of the IL-8 Quotient is not satisfactory enough. However, the PPV and NPV are not intrinsic to the test, but are affected by the prevalence of the disease [36]. To conclude, the results obtained do not equivocally indicate the diagnostic usefulness of the IL-8 Quotient evaluation in the diagnosis of patients with unruptured brain aneurysm.

An intriguing question pertains to a possible mechanism by which cytokines leak to CSF. Obviously, our results gave no unequivocal basis for concluding on this matter, whereas the number of sources in the literature is scarce. Chalouhi et al. [27] evaluated a number of different chemokines and interleukins in blood taken directly from the lumen of unruptured cerebral aneurysm compared to concentration in blood taken from the femoral vessel. The authors observed a significantly higher concentration of selected cytokines, among them IL-8 and MCP-1. As to the source of the cytokines, they suggest that IL-8 and MCP-1 may be released by endothelial cells and by leukocytes (macrophages) infiltrating the foci of endothelial damage [27]. The passive diffusion from the blood seems not to be very probable, considering the relatively large size of these molecules (8–10 kDa) [37]. On the other hand, this cannot be excluded, taking into account the abnormal structure of the aneurismal wall [38].

However, some presumptive evidence indicates also the astroglia and microglia as a local source of chemokines present in the CSF. Both astrocytes and microglia were shown to release IL-8 and MCP-1 when triggered by immune activation, stress and nociceptive input [39,40]. Similar to the brain glia, pericytes can also respond to a range of immunogenic stimuli to induce pro-inflammatory molecules, including tested chemokines [41]. Therefore, the presence of higher levels of the chemokines in the CSF can be alternatively regarded as evidence of an adaptive cerebral immunological reaction in response to ongoing inflammation at a site of aneurysm. At present, this is, however, only a hypothesis, obviously worth of future exploration.

Chalouhi et al. [27] found increased concentration of IL-8 and MCP-1 in blood taken directly from the lumen of cerebral aneurysm [27]. Zhang et al. [28] revealed a significantly higher concentration of MCP-1 chemokine in the blood (plasma) of patients with brain aneurysm compared to healthy people as well as higher MCP-1 levels in patients with multiple aneurysms than in patients with a single aneurysm. The last observation is indirectly consistent with our results, because we also observed a positive correlation between serum MCP-1 and the number of aneurysms. In addition to that, we also found this correlation in the cerebrospinal fluid.

Though general conclusions from the work of Chalouhi et al. and Zhang et al. are rather convergent with ours, some differences in the set-up must also be indicated. First, the two groups of authors conducted their study in mixed populations of patients, containing both ruptured and unruptured aneurysms. This seems important to indicate, as the SAH is a separate pathological entity with serious consequences, also including an aspect of cytokine release, such as cerebral vasospasm [29,42,43]. Admittedly, both groups of authors have separated a subgroup of unruptured aneurysms, but after this maneuver the resulting number of their material became smaller than ours (15 and 10 patients, respectively, in relation to our 25 patients). Other differences include the lack of vertebrobasilar aneurysms in our material and somewhat smaller average size of our aneurisms (5.4 versus 7 mm, median value) compared to the study of Chalouhi et al. Nevertheless, demonstrating the presence of the chemokines as also accompanying smaller aneurysms seems to strengthen the hypothesis of their role in brain aneurysm formation and development.

Chalouhi et al. and Zhang et al. analyzed plasma IL-8 and MCP-1 levels by means of the multiplex method; in our study, the concentration of CSF and serum chemokines was analyzed with the use of the ELISA method. Both the ELISA and multiplex technique use an immobilized antibody to capture the soluble ligand and then detect the captured ligand by a second “reporter” antibody. However, there are significant differences between these two techniques, which include a reporter system, solid phase, or suspension in which the reaction occurs. Determination of the concentration of many ligands simultaneously in the same sample can lead to a cross-reaction (often called “matrix effect”), while ELISA only tests one analyte at a time, thus avoiding such a situation. Moreover, artifacts related to the analysis of many analytes simultaneously can result from: antibodies that can cross-react with other proteins, interspecies antibodies and the presence of other interfering substances [44]. The differences in the level of detection of the multiplex assays probably may translate to discrepancies, e.g., Zhang et al. did not observe significant difference for IL-8.

### Study Limitations

The average size of aneurysms was in our patients was smaller than those in the hitherto published studies. It must be also admitted that patients belonging to our “control group” were not entirely healthy people, as they suffered from persistent facial pain caused by trigeminal nerve irritation. Nevertheless, in spite of these limitations, we still were able to demonstrate statistically significant differences.

CSF samples were not obtained via the lumbar puncture but during craniotomy, so it is not clear if these results are reproducible with CSF lumbar puncture samples. This aspect could also be recognized as a study limitation. However, both unruptured intracranial aneurysm as well as trigeminal neuralgia are not indications for CSF collection for diagnostics purposes, thus the local Bioethics Committee did not give permission for CSF collection via the lumbar puncture. The available literature indicates that the total protein concentration in CSF obtained by lumbar puncture is higher than in CSF obtained from lateral ventricles of the brain. In the case of specific proteins, it is difficult to predict the concentration difference between different space of CSF collection [45,46,47]. Therefore, we suggest that for the interpretation of chemokines concentrations in the CSF, the source of the sample is of crucial importance.

Finally, the setup of our study does not enable us to address the problem of the cellular sources of chemokines elevation in CSF and these specific mechanisms still need to be determined.

## 5. Conclusions

We demonstrated that IL-8 concentration in the cerebrospinal fluid of patients with unruptured intracranial aneurysm is significantly higher than that presented in serum, which can indicate its local synthesis within the central nervous system. CSF IL-8 concentration was also significantly related to aneurysm size, which may reflect the participation of IL-8 in the formation and development of brain aneurysm. We also observed that the Quotient for IL-8 was significantly higher in patients with unruptured intracranial aneurysm compared to those without vascular lesions in the brain. However, further statistical analysis did not clearly indicate the diagnostic usefulness of the IL-8 Quotient evaluation in brain aneurysm patients. Nevertheless, this aspect requires further study.

## Figures and Tables

**Table 1:** Characteristics of unruptured intracranial aneurysm (UIA) patients and control individuals. Results are presented as median with 25th and 75th percentiles. The *p*-value of < 0.05 is considered statistically significant.

	UIA Group	Control Group	*p*-Value
Age (mean, range) (years)	56 (30–70)	57 (25–78)	0.235
Gender: Female/Male	20/5	14/6	0.670
LABORATORY PARAMETERS			
WBC (×10^3^/µL) RBC (×10^6^/µL) HGB (g/dL) HCT (%) PLT (×10^3^/µL) MPV (fL) P-LCR(%) PT (s) INR APTT (s) Fibrinogen (mg/dL)	6.94 (5.31–8.11) 4.52 (4.20–4.80) 13.40 (12.60–14.40) 40.20 (37.60–42.40) 231 (187–246) 11.0 (10.3–11.6) 33.7 (27.1–38.1) 12.2 (11.9–12.5) 0.91 (0.88–0.93) 28.8 (27.3–30.1) 339 (306–391)	6.17 (4.71–7.52) 4.51 (4.12–4.90) 13.45 (12.70–14.90) 40.80 (38.10–43.60) 254 (196–289) 10.8 (10.3–11.1) 30.5 (27.3–33.9) 12.7 (12.5–12.9) 0.99 (0.94–0.99) 26.6 (25.9–29.0) 277 (206–307)	0.195 0.985 0.869 0.742 0.432 0.400 0.371 0.006 0.001 0.034 0.003
Na^+^ (mmol/L)	138 (137–140)	140 (136–140)	0.941
K^+^ (mmol/L)	4.3 (4.0–4.6)	4.5 (4.2–4.7)	0.281
Glucose (mg/dL)	92 (87–102)	89 (86–103)	0.584
Urea (mg/dL)	29 (24–37)	30 (20–49)	0.812
Creatinine (mg/dL)	0.73 (0.66–0.85)	0.78 (0.69–0.83)	0.534
CLINICAL CHARACTERISTICS OF UIA GROUP			
Ethnicity:	All patients belong to Caucasian ethnicity		
Prior SAH:	No	-	-
Number of aneurysm: Single/Multiple	15/10	-	-
Polycyclic aneurysms: *n* (%)	10 (40%)	-	-
Hypertension: *n* (%)	16 (64%)	10 (50%)	0.521
Smoking: *n* (%)	10 (40%)	2 (10%)	0.055
Obesity: *n* (%)	6 (24%)	8 (40%)	0.408

APTT: Activated Partial Thromboplastin, HCT: hematocrit, HGB: hemoglobin, INR: International Normalized Ratio, Time, K^+^: potassium, MPV: mean platelet volume, *n*: number of individuals, Na^+^: sodium, P-LCR: platelet-large cell ratio, PLT: platelet count, PT: prothrombin time, RBC: red blood cell count, SAH: subarachnoid hemorrhage, UIAs: unruptured intracranial aneurysms, WBC: white blood cell count. Conversion factors to SI units are as follows: for WBC: 1.0, for RBC: 1.0, for HGB: 10.0, for PLT: 1.0, for glucose: 0.0555, for creatinine: 88.4.

**Table 2:** Aneurysm geometry characteristics.

No.	Aneurysm Location	Side: L/R	Aneurysm Size ^#^ (mm)	Number of Aneurysms	Polycyclic Aneurysm Yes/No
1.	MCA	R	5.0	4	Yes
2.	MCA	R	6.0	1	No
3.	MCA	L	5.6	2	No
4.	MCA, ICA	R	5.6	6	No
5.	MCA	R	7.0	1	No
6.	MCA	R	5.0	1	No
7.	MCA	L	9.5	1	Yes
8.	ICA	L	3.2	2	Yes
9.	AcomA	R	4.3	3	Yes
10.	MCA	L	4.7	1	No
11.	MCA	L	3.0	1	No
12.	MCA	L	8.0	1	Yes
13.	MCA	L	4.5	2	No
14.	ICA	L	4.0	1	No
15.	ICA	L	4.2	1	Yes
16.	MCA	L	6.0	1	Yes
17.	MCA	L	3.4	1	No
18.	MCA	R	15.0	4	Yes
19.	MCA	L	5.0	1	No
20.	ICA	R	8.0	1	Yes
21.	MCA	R	5.6	1	No
22.	MCA	R	5.4	2	No
23.	MCA	L	7.0	3	No
24.	MCA	R	4.8	1	Yes
25.	MCA	R	7.0	2	No

^#^ The aneurysm’s size is present for the biggest one. MCA: middle cerebral artery, ICA: internal carotid artery, AcomA: anterior communicating artery, L: left, R: right.

**Table 3:** Diagnostic parameters of IL-8 Quotient in differentiating unruptured intracranial aneurysm patients from subjects without intracranial aneurysm.

	Cut-Off	Youden Index	AUC ± SE	Se [%]	Sp [%]	PPV [%]	NPV [%]	ACC [%]	*p*-Value
IL-8 Quotient	2.28	0.42	0.720 ± 0.092	72	70	86	50	71	0.017

Cut-off (based on the highest Youden index); AUC: area under the ROC curve, SE: Standard Error, PPV: positive predictive value, NPV: negative predictive value, ACC: diagnostic accuracy, Se: diagnostic sensitivity, Sp: diagnostic specificity.

**Table 4:** Univariate logistic regression analysis results for UIA diagnosis.

Variable	OR	95%CI	*p*-Value
**Univariate logistic regression analysis**			
IL-8 Quotient	1.84	1.001–3.308	0.050

OR: odds ratio; CI: confidence interval.

**Table 5:** Univariate and multivariate linear regression analysis results for logarithm of the brain aneurysm size.

		**Univariate linear regression analysis**		
**No**	**Variable**	**β**	**e^β^ (95%CI)**	***p*-Value**
1	CSF IL-8 (pg/mL)	0.013	1.013 (1.002–1.024)	0.020
2	BMI (kg/m^2^)	0.034	1.035 (1.004–1.067)	0.029
		**Multivariate linear regression analysis**		
**No**	**Variable**	**β**	**e^β^ (95%CI)**	***p*-Value **
1	CSF IL-8 (pg/mL)	0.014	1.014 (1.003–1.025)	0.014
2	BMI (kg/m^2^)	0.031	1.031 (1.004–1.059)	0.026

β: model coefficient; CI: confidence interval. 3.6. IL-8 and MCP-1 versus aneurysm geometry and risk factors of aneurysm formation.

**Table 6:** CSF and serum IL-8 and MCP-1 concentrations and Quotient values depending on aneurysm’s size median value and aneurysm number and shape.

	Aneurysm Size (mm)		*p*-Value
	<5.4	≥5.4	
CSF IL-8 (pg/mL)	28.2 (22.9–32.5)	39.9 (30.1–44.3)	0.036
Serum IL-8 (pg/mL)	9.6 (8.7–12.1)	11.1 (10.2–16.4)	0.123
IL-8 Quotient	2.5 (2.3–3.4)	3.4 (1.9–5.0)	0.503
CSF MCP-1 (pg/mL)	425.3 (364.1–606.9)	603.6 (378.0–740.0)	0.538
Serum MCP-1 (pg/mL)	238.6 (209.1–359.9)	294.0 (217.6–340.8)	0.852
MCP-1 Quotient	1.9 (1.4–2.0)	1.7 (1.5–2.1)	0.726
	NUMBER OF ANEURYSM		
	Single	Multiple	
CSF IL-8 (pg/mL)	30.9 (23.4–36.9)	31.5 26.2–41.9)	0.461
Serum IL-8 (pg/mL)	9.6 (8.8–11.3)	12.6 (10.2–58.1)	0.062
IL-8 Quotient	3.0 (2.3–3.7)	2.3 (0.7–4.4)	0.397
CSF MCP-1 (pg/mL)	380.0 (362.2–540.6)	650.2 (537.4–746.0)	0.036
Serum MCP-1 (pg/mL)	235.0 (216.6–278.0)	345.6 (294.0–367.4)	0.055
MCP-1 Quotient	1.7 (1.3–1.9)	1.9 (1.7–3.1)	0.367
	POLYCYCLIC ANEURYSMS		
	No	Yes	
CSF IL-8 (pg/mL)	31.7 (24.2–41.6)	30.7 (28.1–36.9)	0.807
Serum IL-8 (pg/mL)	10.2(9.3–16.1)	11.1 (9.6–13.0)	0.892
IL-8 Quotient	2.6 (2.0–3.6)	3.3 1.7–5.0)	0.367
CSF MCP-1 (pg/mL)	427.6 (362.2–712.0)	513.6 (366.2–740.0)	0.765
Serum MCP-1 (pg/mL)	271.6 (230.6–352.4)	249.7 (196.7–312.4)	0.567
MCP-1 Quotient	1.8 (1.4–3.0)	1.7 (1.5–2.7)	0.807

**Table 7:** The optimal cut-off point of CSF IL-8 to predict a larger brain aneurysm size (≥5.4 mm).

	Cut-Off (pg/mL)	Youden Index	AUC ± SE	Se (%)	Sp (%)	PPV (%)	NPV (%)	ACC (%)	*p*-Value
CSF IL-8	36.9	0.615	0.763 ± 0.102	62	100	100	71	80	0.010

Cut-off (based on the highest Youden index), AUC: area under the ROC curve, SE: Standard Error, PPV: positive predictive value, NPV: negative predictive value, ACC: diagnostic accuracy, Se: diagnostic sensitivity, Sp: diagnostic specificity.

**Table 8:** The optimal cut-off point of CSF MCP-1 to predict the number of aneurysms.

	Cut-off (pg/mL)	Youden index	AUC ± SE	Se (%)	Sp (%)	PPV (%)	NPV (%)	ACC (%)	*p*-Value
CSF MCP-1	489.8	0.615	0.753 ± 0.108	90	73	69	92	80	0.019

Cut-off (based on the highest Youden index), AUC: area under the ROC curve, SE: Standard Error, PPV: positive predictive value, NPV: negative predictive value, ACC: diagnostic accuracy, Se: diagnostic sensitivity, Sp: diagnostic specificity.

## Supplementary Materials

The following are available online at https://www.mdpi.com/2077-0383/9/6/1761/s1, Table S1. IL-8 and MCP-1 cerebrospinal fluid (CSF) and serum concentrations and their Quotients in unruptured intracranial aneurysm (UIA) patients compared to control group. Table S2. CSF and serum IL-8 and MCP-1 concentrations and Quotient values depending on aneurysm risk factors: median age value, gender, smoking, hypertension and obesity.
