# Supplementary material for: Ratio of IL-8 in CSF Versus Serum Is Elevated in Patients with Unruptured Brain Aneurysm
Source: J Clin Med. 2020 Jun 5;9(6):1761. doi: 10.3390/jcm9061761 (PMC7356854; doi:10.3390/jcm9061761)
Supplement: Supplementary file 1 [file jcm-09-01761-s001.pdf]

Table S1. IL-8 and MCP-1 cerebrospinal fluid (CSF) and serum concentrations and their Quotients in unruptured intracranial aneurysm (UIA) patients compared to control group.

	UIA GROUP (No.=25)	CONTROL GROUP (No.=20)	*P-value*
	**CSF**		
**IL-8 [pg/ml]**	**31.3** (25.7-39.9)	**26.5** (23.1-33.9)	0.303
**MCP-1 [pg/ml]**	**489.8** (365.8-712.0)	**489.1** (422.8-640.4)	0.957
	**SERUM**		
**IL-8 [pg/ml]**	**10.9** (9.3-13.0)	**16.5** (11.4-33.2)	0.065
**MCP-1 [pg/ml]**	**271.6** (217.6-350.4)	**314.7** (268.0-382.6)	0.240
	**Quotient**		
**IL-8 Quotient**	**2.83** (1.97-3.72)	**1.55** (0.77-2.70)	***0.045***
**MCP-1 Quotient**	**1.77** (1.45-2.03)	**1.62** (1.20-2.01)	0.418

Table S2. CSF and serum IL-8 and MCP-1 concentrations and Quotient values depending on aneurysm risk factors: median age value, gender, smoking, hypertension and obesity.

	**AGE**		***P*-value**
	**< 60**	**≥ 60**	
**CSF IL-8 [pg/ml]**	**31.7** (26.2-39.9)	**30.5** (20.6-41.6)	0.461
**Serum IL-8 [pg/ml]**	**11.0** (9.6-16.1)	**9.9** (8.8-12.5)	0.461
**IL-8 Quotient**	**2.8** (1.9-4.0)	**2.7** (2.0-3.4)	0.723
**CSF MCP-1 [pg/ml]**	**537.4** (362.4-712.0)	**408.2** (365.8-740.0)	0.807
**Serum MCP-1 [pg/ml]**	**271.6** (221.4-367.4)	**265.3** (216.6-312.4)	0.397
**MCP-1 Quotient**	**1.7** (1.5-2.0)	**1.9** (1.4-2.3)	0.683
	**GENDER**		
	**Female**	**Male**	
**CSF IL-8 [pg/ml]**	**30.5** (23.8-40.7)	**36.7** (31.3-36.9)	0.488
**Serum IL-8 [pg/ml]**	**10.2** (9.3-12.1)	**12.5** (12.2-16.01)	0.272
**IL-8 Quotient**	**2.7** (2.0-3.9)	**3.0** (2.3-3.4)	0.767
**CSF MCP-1 [pg/ml]**	**425.3** (364.21-638.4)	**712.0** (627.2-740.0)	0.216
**Serum MCP-1 [pg/ml]**	**262.1** (217.1-352.6)	**278.0** (235.0-294.0)	0.869
**MCP-1 Quotient**	**1.7** (1.4-1.9)	**2.1** (2.0-2.7)	0.083
	**SMOKING**		
	**No**	**Yes**	
**CSF IL-8 [pg/ml]**	**31.7** (25.7-39.9)	**29.6** (24.2-41.9)	0.849
**Serum IL-8 [pg/ml]**	**11.3** (9.6-16.4)	**9.7** (8.8-10.9)	0.129
**IL-8 Quotient**	**2.5** (1.7-4.0)	**3.0** 2.3-3.7)	0.461
**CSF MCP-1 [pg/ml]**	**603.6** (362.4-761.4)	**458.7** (365.8-540.6)	0.428
**Serum MCP-1 [pg/ml]**	**271.6** 216.6-350.4)	**373.3** (230.6-352.4)	0.849
**MCP-1 Quotient**	**1.8** (1.4-2.3)	**1.7** (1.5-2.0)	0.567
	**HYPERTENSION**		
	**No**	**Yes**	
**CSF IL-8 [pg/ml]**	**30.1** (25.7-31.7)	**36.2** (24.8-41.7)	0.419
**Serum IL-8 [pg/ml]**	**11.0 (**8.6-16.4)	**10.6** 9.6-12.3)	0.978
**IL-8 Quotient**	**2.8** (1.7-4.0)	**2.8** (2.3-3.7)	0.637
**CSF MCP-1 [pg/ml]**	**673.2** (540.6-761.4)	**373.1** (349.3-558.5)	***0.005***
**Serum MCP-1 [pg/ml]**	**350.4** (230.6-375.0)	**262.1** (207.2-304.6)	0.169
**MCP-1 Quotient**	**1.8** (1.7-2.3)	**1.7** (1.4-2.0)	0.229
	**OBESITY**		
	**BMI < 29.99**	**BMI ≥ 30.00**	
**CSF IL-8 [pg/ml]**	**31.7** (24.2-41.6)	**30.7** (28.1-36.9)	0.926
**Serum IL-8 [pg/ml]**	**10.9** (9.3-16.1)	**10.5** (9.6-16.1)	0.598
**IL-8 Quotient**	**2.6** (1.9-3.7)	**3.7** (2.3-5.0)	0.247
**CSF MCP-1 [pg/ml]**	**427.6** (362.2-673.2)	**614.9** (380.0-761.4)	0.303
**Serum MCP-1 [pg/ml]**	**271.6** (221.4-3652.8)	**247.3** (196.7-296.8)	0.333
**MCP-1 Quotient**	**1.7** (1.4-2.0)	**2.3** (1.7-3.5)	0.138
